# Dimple‐Encoded Reprogrammable Origami

**DOI:** 10.1002/advs.76901

**Published:** 2026-08-05

**Authors:** Qun Zhang, Weicheng Huang, Amir Hajiyavand, Hyunyoung Kim, Claire Dancer, Karl Dearn, Mingchao Liu

**Affiliations:** ^1^ Department of Mechanical Engineering University of Birmingham Birmingham UK; ^2^ School of Engineering Newcastle University Newcastle upon Tyne UK; ^3^ School of Computer Science University of Birmingham Birmingham UK; ^4^ School of Metallurgy and Materials University of Birmingham Birmingham UK

**Keywords:** bistable dimple, morphing structure, origami mechanism, programmable folding

## Abstract

Programmable folding of elastic sheets typically relies on predefined flexible creases or active materials‐enabled hinges, which lack intrinsic bistability and limit reprogrammability within a single structure. Here, we present a dimple‐encoded origami platform that converts bistable dimple snapping into dimple‐encoded hinge bands with prescribed folding angles in a continuous sheet. This interaction‐enabled mechanism enables the design of distributed hinge networks through the arrangement and selective inversion of dimples. We establish folding‐angle design charts that can be directly used to select local dimple arrangements for a target folding angle, forming a practical hinge‐angle library without altering the underlying unit geometry. Using this approach, a single dimpled sheet can be reprogrammed to realize multiple distinct configurations, such as triangle, square, and pentagon shapes. We further extend the method to flat‐to‐3D morphing of polyhedral origami and validate the results through experiments and finite element simulations. As demonstrations, we realize self‐supporting cubic shells with enhanced impact resistance and partially deployable cube configurations that remain stable upon opening, highlighting their potential for protective enclosures and deployable architectural structures. The proposed strategy provides a fabrication‐friendly route to reprogrammable shape‐morphing and adaptive mechanical systems.

## Graphical abstract

Local snap‐through of bistable dimples turns flat elastic sheets into reprogrammable origami structures. Interaction‐guided dimple arrays act as angle‐programmable hinge bands, enabling one sheet to morph into distinct polygonal and three‐dimensional configurations while retaining stable shapes for deployable and protective functions.

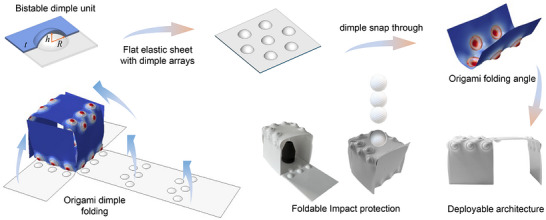

## 1 Introduction

Transforming planar sheets into three‐dimensional (3D) structures through origami‐inspired design has become a promising paradigm for creating deployable devices, reconfigurable metamaterials, and shape‐morphing systems across a wide range of length scales [1, 2, 3]. By encoding geometry into a planar sheet, complex 3D morphologies and programmable mechanical functions can be generated through structural transformation, reducing the need for motors, bulky mechanisms, and heavy rigid assemblies. Such an approach offers lightweight solutions for robotics, wearable systems, biomedical devices, aerospace deployables, and protective packaging [4, 5, 6, 7].

A central requirement in these applications is the ability to control folding behavior through hinge design, namely to prescribe both the location and the magnitude of folding in a predictable and robust manner [8, 9, 10, 11]. In most existing approaches, hinges are realized through predefined creases, compliant regions, or multi‐material architectures that impose fixed folding pathways [12, 13]. As a result, the folding behavior is largely determined at the fabrication stage, and a given sheet can typically realize only a limited set of predesigned folded configuration.

Multistability offers a route to overcome this limitation by allowing structures to switch between distinct stable states and retain their configurations without continuous energy input [14]. In this context, bistable elements provide a particularly simple and widely used realization: they possess two stable equilibria separated by an energy barrier, enabling reconfiguration through snap‐through [15, 16]. Bistability arises in a wide variety of elastic structures, ranging from beams [17], rods [18], and ribbons [19] to shells [20]. Among these, thin‐shell elements, such as spherical dimples or domes, serve as canonical bistable units because they can be inverted between two stable configurations and retain either state due to geometric constraints [21]. Arrays of such elements have therefore been widely explored for morphable sheets, reconfigurable architectures, and mechanical metamaterials [22, 23, 24, 25].

In many origami systems, multistability arises from global geometric constraints, leading to multiple stable configurations at the structural level [26, 27, 28, 29]. Beyond conventional crease‐based origami, locally bistable mechanisms have also been used to realize programmable global morphing and tunable mechanical responses in architected sheets and metamaterials [30, 31]. In addition, global multistability in origami has recently been achieved by integrating locally bistable creases [32]. Together, these studies establish local bistability as an effective design principle for reconfiguration.

However, for origami‐style shape programming, two related challenges remain: one is to convert interacting bistable units into reliable hinge‐like building blocks with prescribed and predictable folding angles; the other is to compose these local building blocks within a single continuous sheet so that different global configurations can be accessed in a programmable manner. Although selective inversion of bistable domes can program global morphing and mechanical responses, a calibrated mapping from dimple‐array interaction parameters to a prescribed local retained folding angle remains limited. Such a mapping is required to transform interacting dimple arrays from distributed morphing elements into composable hinge bands for reprogrammable origami architectures.

**FIGURE 1:**
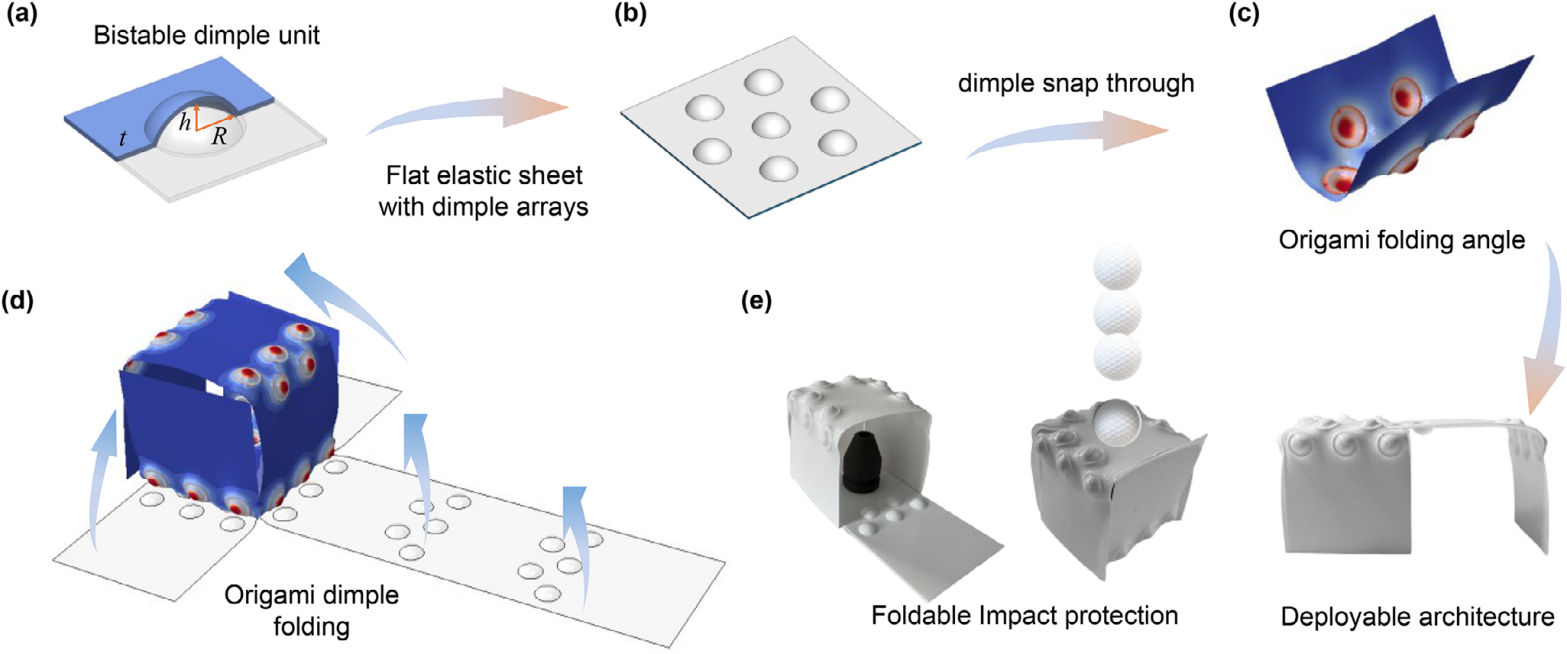
Dimple‐encoded reprogrammable origami: concept and design principle. (a) A bistable dimple unit defined by geometric parameters (t, h, R). (b) Arrays of dimples embedded in a flat sheet. (c) Interactions between neighboring snapped dimples generate localized dimple‐encoded hinge bands, defining a characteristic folding angle. (d) These interaction‐induced hinges can be spatially organized through the placement and selective inversion of dimples to guide origami‐like folding of the sheet. (e) The resulting dimple‐encoded origami structure enables reprogrammable shape‐morphing, allowing different stable target configurations to be achieved from the same sheet, with potential applications in deployable and adaptive structures.

Here, we introduce a dimple‐encoded multistable origami platform in which interacting bistable dimples form localized dimple‐encoded hinge bands, defined here as finite‐width dimple‐patterned regions that localize bending and function as effective hinges after dimple inversion, with programmable folding angles (Figure 1). By keeping the individual dimple geometry fixed and varying the array topology together with the nondimensional longitudinal and transverse spacings, we establish a calibrated relationship between dimple–dimple interactions and the retained local folding angle. The resulting hinge‐angle library is then used to compose multi‐hinge strips and planar nets, enabling reprogrammable polygonal frames and flat‐to‐3D polyhedral shells.

Overall, this work establishes a design framework that leverages interactions between local bistable elements to generate programmable hinge networks with predictable folding, providing a scalable and fabrication‐friendly route toward reprogrammable morphing systems and deployable architectures.

## 2 Results and Discussion

### 2.1 Dimple‐Encoded Origami: Concept and Interaction‐Enabled Hinges

We begin by introducing the concept of dimple‐encoded origami, in which bistable dimples embedded in a continuous sheet are used to define spatially arranged hinge bands (Figure 1). Each dimple acts as a local bistable unit that can be selectively inverted, generating localized deformation in the surrounding sheet (Figure 1a). When multiple dimples are arranged in proximity, their interactions give rise to effective hinge‐like behavior, enabling controlled folding along prescribed directions (Figure 1b,c).

This interaction‐enabled mechanism provides a route to encode folding behavior directly into the sheet through the spatial arrangement and activation of dimples (Figure 1d). While several prior studies have demonstrated reconfigurable hinge properties, global morphologies, and mechanical responses by switching locally bistable mechanisms [23, 24, 31, 32], the present strategy focuses on using interacting bistable dimples as angle‐programmable hinge bands. In this framework, the local retained folding angle is governed by the combined effects of dimple topology, spacing, and activation state, providing a quantitative route to prescribe hinge‐like folding within a continuous sheet.

Collectively, these interaction‐enabled mechanisms allow bistable dimples to be organized into spatially distributed arrays whose selective activation defines programmable hinge networks within a continuous sheet. By composing angle‐programmable hinge bands in this manner, a single sheet can access multiple distinct stable folded configurations, enabling controlled global shape transformations and reconfiguration between stable states using the same structure.

Beyond geometric morphing, such interaction‐enabled hinge networks provide a foundation for functional systems, including deployable enclosures and spatial structures that remain stable without continuous external support (Figure 1e). These capabilities are explored in the following sections through quantitative design rules and representative demonstrations.

### 2.2 Interaction‐Guided Folding and Angle Design

**FIGURE 2:**
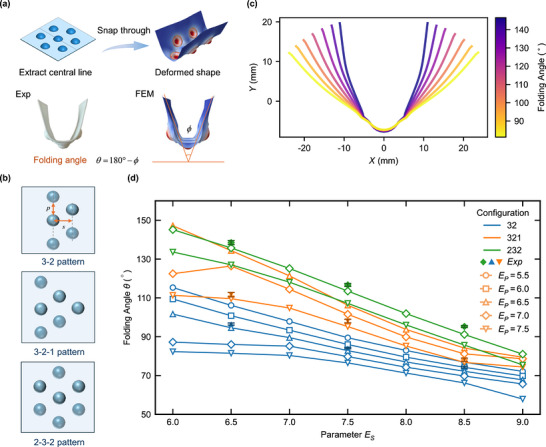
Dimple interaction‐enabled hinges and folding‐angle design charts. (a) Extraction of the hinge opening angle ϕ from the centerline of the deformed dimpled sheet, and the definition of the folding angle $\theta =180^{\circ }-\phi$. (b) Definitions of the longitudinal pitch p and transverse spacing s (nondimensionalized as $E_{p}=p/l_{p}$ and $E_{s}=s/l_{p}$), and representative array topologies (‘3–2’, ‘3–2–1’, and ‘2–3–2'). (c) Representative deformed centerlines corresponding to different folding angles for a fixed array configuration (2–3–2 pattern) and representative parameters. (d) Design charts showing the folding angle θ as a function of $E_{s}$ for different values of $E_{p}$ and array configurations.

As illustrated in Figure 2a, hinge‐like folding can be induced by arranging multiple bistable dimples within a continuous sheet and selectively activating them through inversion. Interactions between neighboring snapped dimples give rise to localized hinge bands, characterized by a well‐defined folding angle θ.

The key to programmable hinge behavior therefore lies in controlling these dimple–dimple interactions. When a dimple snaps, the induced deformation localizes within a narrow region where bending and stretching compete [24], introducing a natural interaction length scale that governs how nearby dimples couple.

A simple scaling argument captures this characteristic length. The competition between bending, which favors smooth curvature, and stretching, which penalizes in‐plane deformation, leads to a localized boundary layer with a characteristic size [24]

(1) $$\ell_{b}∼\sqrt{tR},$$

where t is the sheet thickness and R is the dimple radius. This characteristic length, also referred to as the Pogorelov length scale [33, 34], provides a physical measure of the interaction range: strong coupling occurs when spacings are comparable to $\ell_{b}$, whereas weak coupling arises when dimples are well separated.

Based on this interaction length, we parameterize dimple arrays using the longitudinal pitch p and transverse spacing s (Figure 2b), normalized as

(2) $$E_{p}=\frac{p}{\ell_{b}},\;E_{s}=\frac{s}{\ell_{b}}.$$

This nondimensional description collapses geometric effects into a compact interaction‐based design space, enabling different array topologies to be compared on equal footing.

In constructing the hinge‐angle design charts, the single‐dimple geometry was fixed at radius R=5 mm and height h=3.5 mm, corresponding to h/R=0.7, together with a fixed sheet thickness t=0.6 mm and fixed material properties. This baseline geometry was selected based on the single‐dimple bistability screening (Figure S1) and practical fabrication considerations. With the intrinsic dimple geometry fixed, variations in the folding angle can be attributed primarily to changes in dimple–dimple interaction, controlled by the normalized spacings $E_{p}$ and $E_{s}$ and by the array topology.

Since a single column of dimples fails to produce well‐defined and controllable folding (Figure S2), we consider three representative multi‐column patterns, namely “3–2,” “3–2–1,” and “2–3–2,” as shown in Figure 2b. Each pattern is embedded in a square sheet with dimensions of 60 mm × 60 mm. The corresponding design spaces for each pattern, in which all snapped dimples remain bistable and the resulting deformation localizes into a well‐defined hinge, are mapped in Figure S3. These three patterns are intended as representative multi‐column hinge building blocks for establishing the interaction‐guided design workflow, rather than as an exhaustive library of all possible dimple arrangements. Additional hinge topologies can be incorporated by applying the same calibration procedure, and the design claims in this work are therefore restricted to the validated topologies and bistable design domains reported here.

Using the folding angle θ extracted from the deformed configurations obtained from finite element method (FEM) simulations (Figure 2a,c), we construct angle design charts that map $\left(E_{p},E_{s}\right)$ to the resulting hinge response for each topology (Figure 2d). Across all configurations, two consistent trends emerge: increasing either $E_{s}$ or $E_{p}$ weakens dimple–dimple coupling and leads to shallower folds, corresponding to smaller θ, reflecting reduced interaction‐induced incompatibility.

To further validate the accuracy of the extracted folding angles, we performed independent measurements on 3D‐printed samples using the OpenCV‐based contour extraction and TLS line‐fitting method (Figure S4). Three groups of samples were fabricated according to the three patterns introduced before, and tested under the same activation and relaxation protocol (Figure S5). The experimentally measured folding angles are plotted in Figure 2d as solid symbols, showing good agreement with the FEM‐based design charts. This agreement supports the reliability of the angle‐extraction workflow and confirms that the simulated design charts can be used as a practical hinge‐angle library for programmable folding.

We further examined the sensitivity of the final folded state to the order of manual dimple activation. As shown in Movie S1, for the representative hinge patterns tested here, activating the same target set of dimples in different sequences leads to the same relaxed folded configuration. This observation supports that, within the validated robust bistable bending domain, the programmed hinge folding state is primarily governed by the activated dimple set and array geometry rather than by the detailed triggering order.

The two spacing directions and the hinge topology play distinct yet complementary roles in design. The transverse spacing $E_{s}$ governs cross‐column coupling and can induce qualitative changes in deformation mode, often accompanied by distributed bending or warping (Figure S3), whereas the longitudinal pitch $E_{p}$ provides a continuous and predictable parameter for tuning the folding angle within a given mode. The hinge topology further sets the accessible angle range: the “3–2” configuration yields smaller folding angles, whereas “3–2–1” and “2–3–2” enable larger θ and a wider range, particularly at smaller $E_{s}$. At larger $E_{s}$, the responses of different topologies converge, consistent with weakened coupling in the weak‐interaction regime. These results establish a practical design strategy: $E_{s}$ and topology are first selected to ensure a stable deformation mode, after which $E_{p}$ is used to continuously tune the target folding angle.

It is worth noting that the hinge‐angle design discussed above applies to activated dimple states. When the dimples are not activated, the inversion‐induced geometric incompatibility is absent. The sheet may still bend elastically under external loading, but such deformation does not constitute a programmed localized hinge state and is not expected to retain a prescribed folding angle after unloading. In addition, in the current single‐sided dimple design, the retained folding direction is selected by the direction of dimple inversion; stable folding in the opposite direction would require additional strategies, such as activation from the opposite face or a symmetric/double‐sided dimple architecture.

Taken together, these results establish a geometry‐driven design framework for programmable hinge behavior. The resulting angle design charts (Figure 2d) thus function as a practical hinge‐angle library, enabling prescribed folding angles to be achieved without modifying the underlying bistable units.

### 2.3 Reprogrammable Multi‐Hinge Origami Structures

With the folding angle design charts established, we next demonstrate how these calibrated hinges can be composed to program system‐level origami morphing. The key idea is to treat a single strip as a sequence of quasi‐rigid segments connected by localized dimple‐encoded hinge bands, where each hinge band is assigned a prescribed folding angle by selecting its local array topology and interaction parameters $\left(E_{p},E_{s}\right)$ from the hinge‐angle library given in Section 2.2.

**FIGURE 3:**
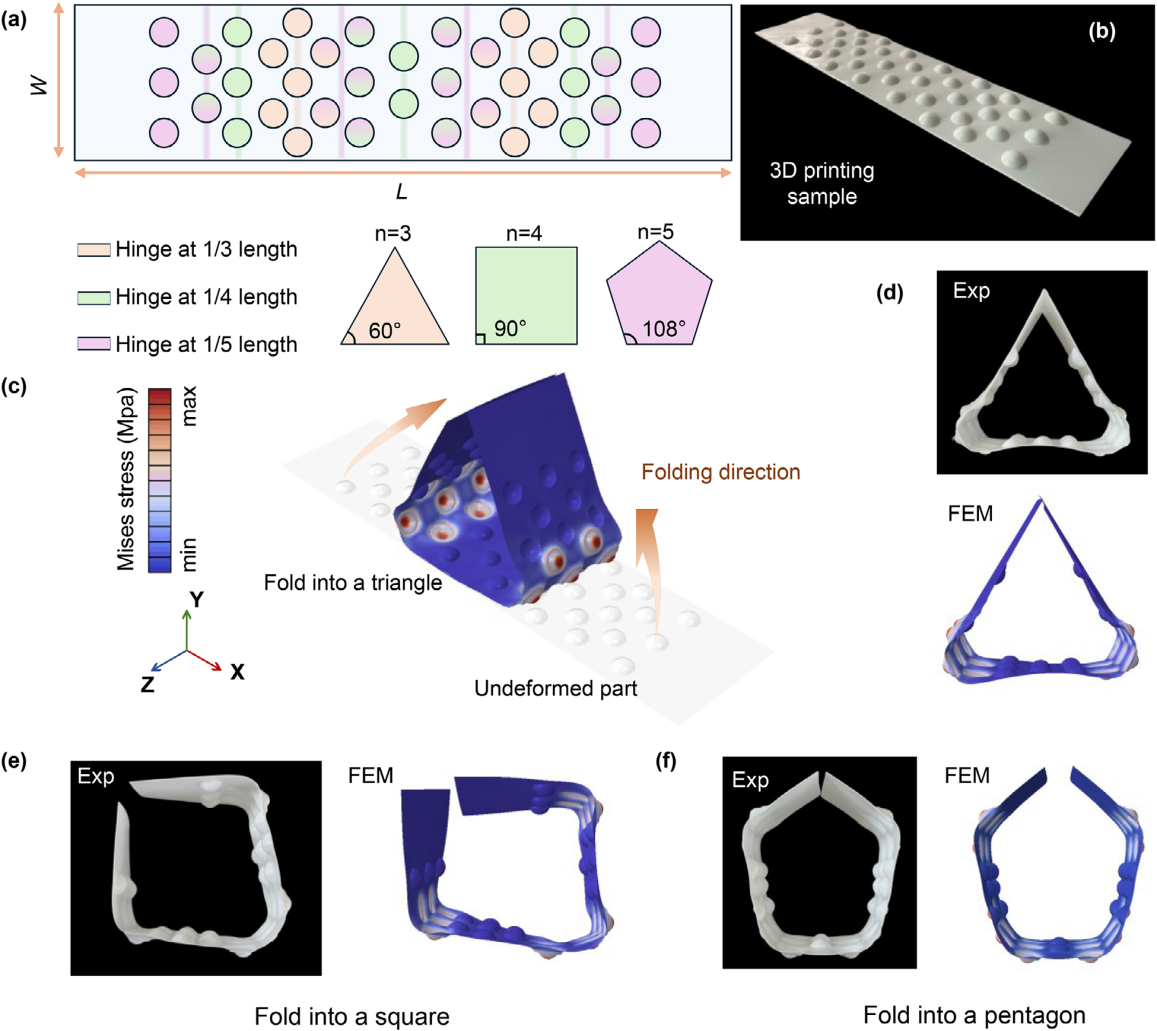
Dimple‐encoded multi‐hinge strip for reprogrammable shape‐morphing. (a) (Top) Schematic of a single elastic strip patterned with bistable dimple arrays. The colored bands indicate localized dimple‐encoded hinge bands placed at prescribed locations along the strip for different target polygonal configurations. (Bottom) Corresponding hinge‐placement rule for regular polygonal shapes, where the required turning angle is the exterior angle $\psi_{n}=360^{\circ }/n$ (triangle: $120^{\circ }$, square: $90^{\circ }$, pentagon: $72^{\circ }$; the corresponding interior angles are $60^{\circ }$, $90^{\circ }$, and $108^{\circ }$). (b) 3D‐printed prototype of the dimple‐encoded strip. (c) Demonstration of the folding process into an equilateral triangle through FEM simulation. (d) Folding into an equilateral triangle, with comparison between experimental realization (Exp) and numerical prediction (FEM). (e) Reconfiguration of the same strip into a square. (f) Reconfiguration into a regular pentagon, further demonstrating reprogrammable shape‐morphing within a single structure.

Figure 3 illustrates the design and implementation of a single strip that can be reprogrammed into multiple target shapes. In the strip layout, colored bands indicate the hinge locations, each encoding a distinct target turning angle (Figure 3a). For a regular n‐gon frame, the turning angle at each vertex is given by the exterior angle [35]:

(3) $$\psi_{n}=\frac{360^{\circ }}{n},$$

which can be realized by assigning hinges with $\theta \approx \psi_{n}$ using the calibrated $\left(E_{p},E_{s}\right)$–θ relation. Notably, this programming is achieved without altering the bistable unit geometry: only the local spacing and topology are varied, making the approach fabrication‐friendly and scalable.The elastic strip used in both experiments and simulations has dimensions of length L=250 mm and width W=60 mm.

Using this workflow, multiple hinge sets are encoded along the same strip to target different polygonal configurations. A representative strip is fabricated by 3D printing, as shown in Figure 3b. As demonstrated by the FEM simulation in Figure 3c and Movie S2, snapping selected dimples activates the hinges located at one‐third of the strip length, thereby folding the strip into a triangular configuration.

Furthermore, as shown in Figure 3d–f, the same strip can be reconfigured into triangular, square, and pentagonal configurations by selectively inverting different subsets of dimples. Experiments and FEM simulations show good qualitative agreement, confirming that the interaction‐guided hinge‐angle library enables predictable and repeatable reprogramming within a single structure. Each configuration is maintained as a stable state without external constraint due to the intrinsic bistability of the activated dimple arrays. The corresponding experimental reconfiguration process is shown in Movie S3, where the strip is sequentially reconfigured into these polygonal configurations and remains stable after each reconfiguration.

Two considerations are important for reliable composition. First, hinge segments must operate within the bistable regime to avoid relaxation and unintended spring‐back (Figure S3). Second, neighboring hinge bands must be sufficiently separated to avoid overlap of adjacent deformation boundary layers, which may otherwise distort the prescribed angles and accumulate geometric error. Accordingly, the present hinge‐angle library should be interpreted as a calibrated design guideline for well‐separated hinge bands, rather than as a universal independent‐superposition rule for arbitrarily dense hinge networks.

Together, these results demonstrate that the interaction‐guided hinge‐angle library is not only descriptive but also constructive: it enables distributed angle encoding along a single structure to achieve multiple distinct configurations on demand. In the next section, we extend this concept from planar frames to fully three‐dimensional polyhedral origami shells.

### 2.4 Flat‐to‐3D Polyhedral Origami

Having established reprogrammable folding within planar strips, we next demonstrate that the same interaction‐guided hinge‐angle library can be extended to construct fully three‐dimensional origami structures from a flat sheet. The central idea is to encode spatially distributed hinges directly into a planar net such that the target polyhedral geometry emerges upon activation, without the need for discrete joints, multi‐material interfaces, or external assembly.

Our approach is constructive. A target polyhedron is first unfolded into a planar net, and each fold line is assigned a localized dimple‐encoded hinge with a prescribed folding angle selected from the design charts. The faces remain comparatively rigid, while deformation is confined to the programmed hinge bands, allowing the global three‐dimensional shape to be dictated entirely by local geometry. In this way, complex shell structures can be realized through a single‐material, geometry‐driven design framework.

**FIGURE 4:**
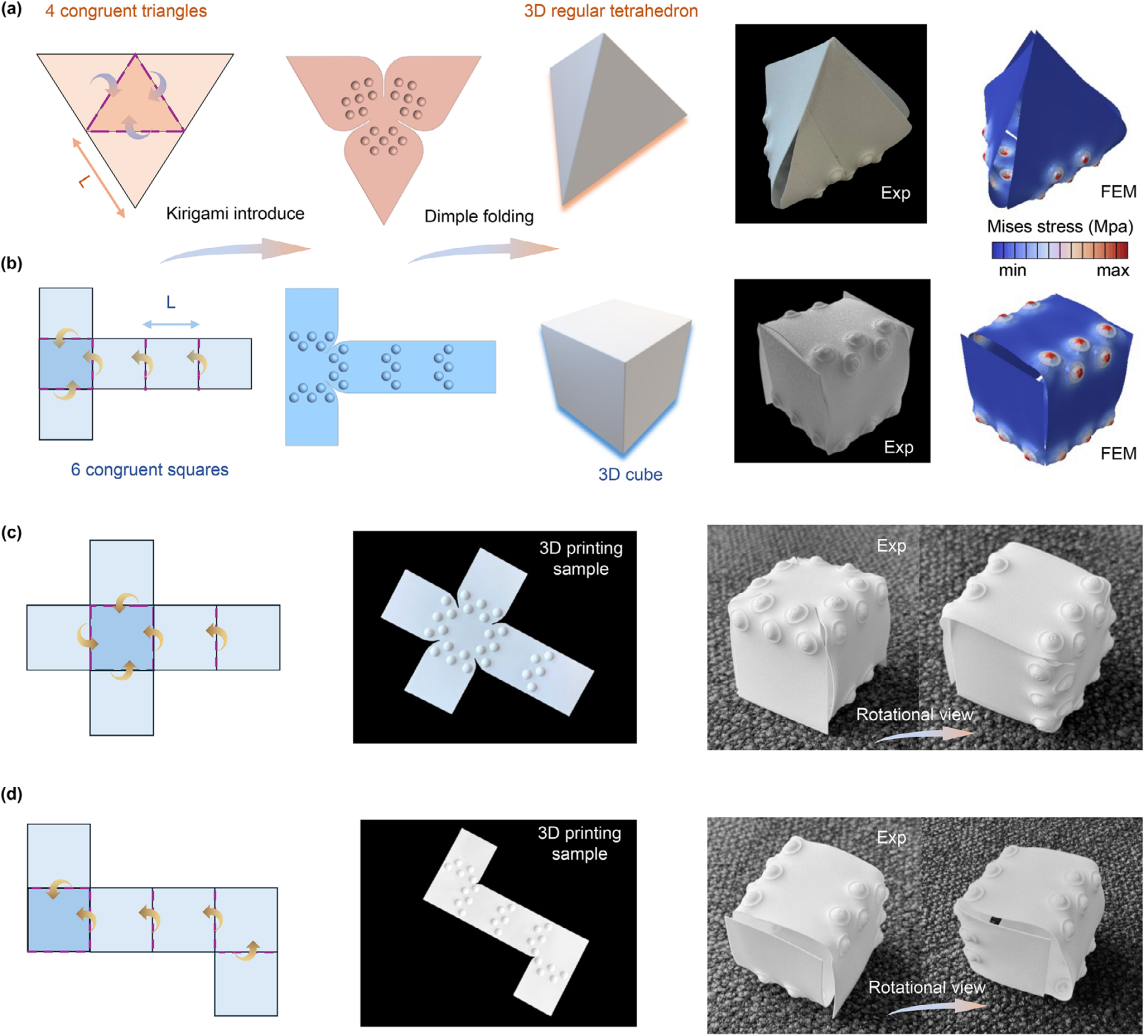
Flat‐to‐3D polyhedral origami shells programmed by dimple‐encoded hinges. (a) Planar net of four congruent triangles, where dimple‐encoded hinges with angles selected from the calibrated hinge‐angle library (Figure 2) are encoded and kirigami cuts are introduced to enable folding into a regular tetrahedron; experimental realization (Exp) and numerical prediction (FEM) are shown for comparison and demonstrate agreement in the assembled shell and deformation localization near hinge bands. (b) Planar net of six congruent squares folds into a cube, with validation from both Exp and FEM results. (c,d) Alternative cube net layouts fabricated by 3D printing and their corresponding folded cube enclosures in experiments, shown in different rotational views, illustrating that different planar layouts can realize the same target polyhedron under the same hinge‐selection workflow.

Figure 4 illustrates this concept through representative demonstrations. A net composed of four congruent triangular panels folds into a regular tetrahedron (Figure 4a), providing a minimal example that validates the generality of the approach across different folding angles. Kirigami cuts are introduced at the hinge edges to suppress out‐of‐plane warping [36] (Figure S6). In the flat‐to‐3D folding experiments and simulations, the planar net of the regular tetrahedron consists of four congruent equilateral triangles with a side length of L=120 mm, resulting in a total footprint length of 240 mm. For the hexahedron (3D cube), the planar layout comprises six congruent squares, each characterized by a side length of L=60 mm. Next, a net of six square panels is programmed to assemble into a cubic shell, forming a robust, self‐supporting enclosure (Figure 4b). 3D‐printed experiments and FEM simulations show consistent folding pathways and final geometries, with deformation strongly localized at the hinge bands, thereby confirming the predictive capability of the hinge‐angle library. The folding processes of the tetrahedral and cubic structures are shown in FEM simulations in Movie S4 and in experiments on 3D‐printed samples in Movie S5.

Beyond geometric assembly, this platform enables functional three‐dimensional systems. The resulting polyhedral shells are intrinsically stable due to the bistability of the hinges, allowing them to maintain their shape without continuous external input. Moreover, different planar nets leading to the same target polyhedron can be encoded within the same design framework (Figure 4c,d), offering flexibility in deployment pathways and accessible intermediate configurations. This ability to program both the final shape and the transformation pathway highlights the potential of dimple‐encoded origami for deployable enclosures, protective structures, and scalable architectural systems. From a design perspective, the planar net introduces an additional programming dimension beyond hinge‐angle selection: while the hinge‐angle library prescribes local folding angles, the net geometry governs the global folding pathway and determines the set of accessible configurations and functional states.

These results demonstrate that the interaction‐guided hinge‐angle library extends naturally from planar morphing to spatial assembly, providing a unified route to fabricate complex, self‐supporting three‐dimensional origami structures from a single sheet.

We note that the present demonstrations focus on hinge‐band composition in polyhedral nets, where vertex coupling remains relatively limited. Extending the framework to higher‐degree origami vertices, such as Miura‐ori patterns, would require additional vertex‐level design because multiple folding angles are geometrically coupled and cannot be independently prescribed by the current one‐dimensional hinge library. A possible extension is to treat such vertices as distinct two‐dimensional building blocks, potentially combined with localized kirigami releases or dimple‐free vertex regions to reduce hinge–hinge coupling.

### 2.5 Functional Demonstrations: Impact Protection and Deployable Spatial Structures

**FIGURE 5:**
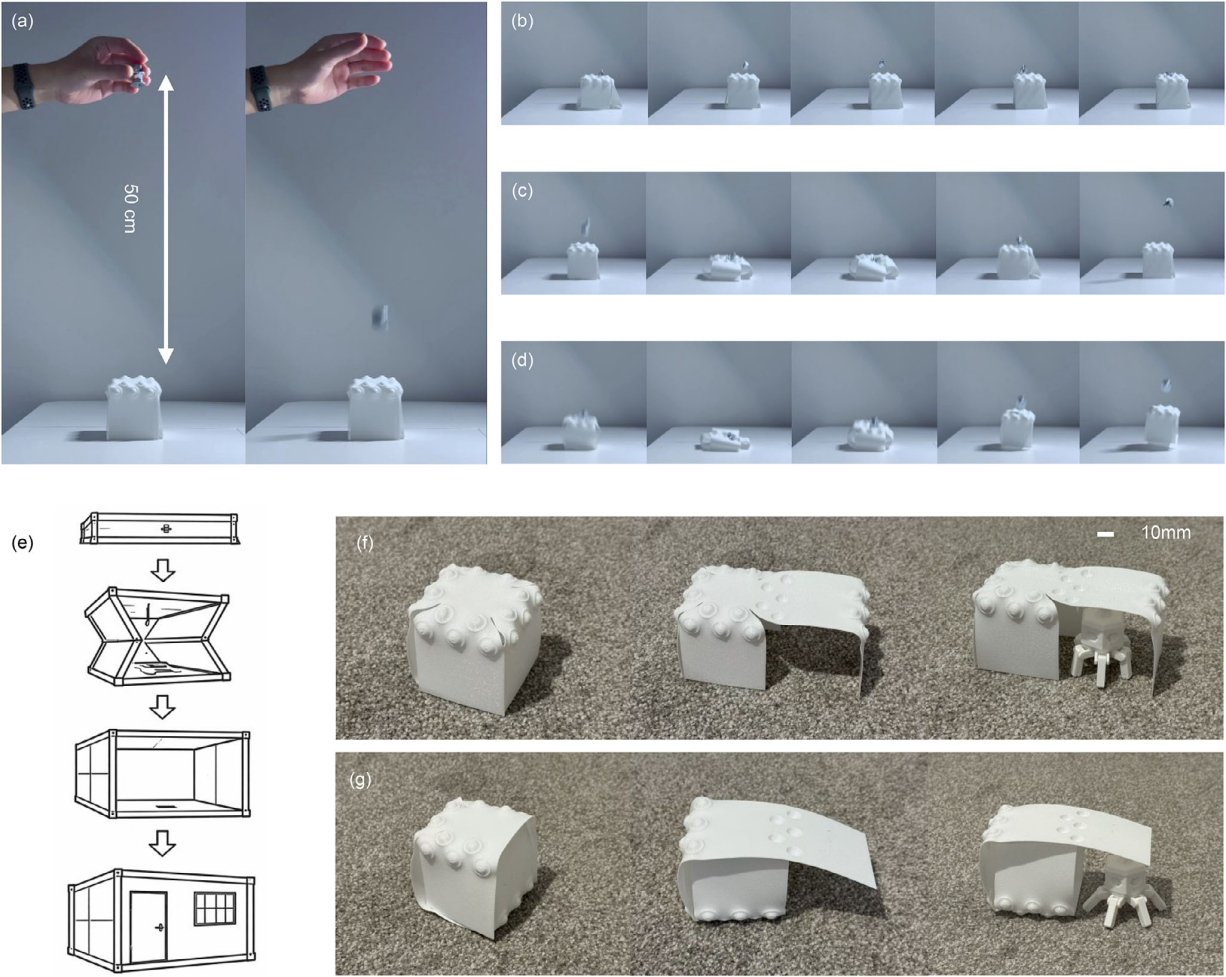
Functional demonstrations of dimple‐encoded deployable origami cubes: impact protection and scalable spatial configurations. (a) Schematic of the drop‐impact test. (b–d) Impact‐protection demonstration: sequential frames from a single drop‐impact experiment showing the response of the pre‐folded origami cube under increasing impact energies. The structure deforms upon impact while maintaining an enclosed volume, thereby protecting the payload. (e) Schematic illustration of a deployable architectural concept, highlighting multiple stable states from fully enclosed to partially deployed configurations. (f) Partially deployed configuration forming a stable and accessible spatial structure. (g) Alternative partially deployed configuration illustrating net‐dependent geometries for deployable architectural applications.

The dimple‐encoded origami serves not only as a geometric demonstration but also as a functional structure with intrinsic multistability and reprogrammability, whose behavior can be tailored through the choice of planar net and folding pathway. Figure 5 summarizes two complementary functionalities enabled by the same dimple‐programmed origami cube.

First, the origami cube provides impact protection for delicate payloads [37]. A payload placed inside the folded cube remains shielded under controlled drop/impact tests (Figure 5a–d and Movie S6; details in the Experimental Section), demonstrating that the bistable, dimple‐encoded origami shell can maintain a protective cavity and mitigate impact transmission. This protection arises from two coupled mechanisms: the closed polyhedral geometry distributes loads across multiple faces and hinges, avoiding localized failure, while the bistable dimple‐encoded hinges provide local buffering through deformation, reducing direct load transmission to the payload. Notably, as the impact load increases from Figure 5b–d, the resulting deformation becomes progressively larger; however, in all cases, the structure fully recovers its original shape without external intervention, demonstrating its impact resilience. To provide quantitative support for this impact‐buffering response, we further extracted video‐based impact metrics, including the first‐bounce rebound height $H_{r}$, maximum compression displacement $\delta_{\max}$, height‐based apparent coefficient of restitution $e_{\mathrm{app}}$, and rebound‐energy‐loss proxy $\Delta E_{\mathrm{proxy}}$ (Table S1 and Figure S7). These measurements show that the origami cube deformation increases with impactor mass, providing quantitative evidence for recoverable structural deformation during impact.

Second, the dimple‐encoded origami structure provides a model system for deployable architectural designs [38] (Figure 5e). As illustrated in Figure 5f,g, the folded cube can transition between fully enclosed and partially deployed configurations through the selective inversion of bistable dimples, with each state remaining intrinsically stable without external support. This multistability enables controllable spatial reconfiguration within a single structure, allowing the degree of enclosure to be actively reprogrammed. Although demonstrated at a small scale, the underlying design principles are inherently scalable, suggesting a pathway toward geometry‐driven, reconfigurable architectural structures while requiring further advances in manufacturing and materials for large‐scale implementation.

Together, these results demonstrate that interaction‐guided bistable dimples constitute a constructive design language: calibrated local hinge behavior can be composed to program global flat‐to‐3D assembly, while net geometry enables functional adaptability within a single deployable system.

## 3 Conclusion

In summary, we developed a dimple‐encoded bistable origami platform that converts snap‐through of a single bistable unit into dimple‐encoded hinge bands with programmable folding angles in a continuous sheet. By exploiting the interaction length scale associated with shallow‐shell boundary‐layer localization, we introduced a compact nondimensional design space for dimple coupling and established a robust folding‐angle metric, enabling quantitative angle design charts that can be directly inverted to select local dimple arrangements for prescribed folding responses. This interaction‐guided framework elevates bistable dimples from a qualitative morphing mechanism to a predictive and constructive design tool. Using this approach, we demonstrated reprogrammable multi‐hinge strips capable of accessing multiple polygonal configurations within a single structure, and extended the same strategy to flat‐to‐3D assembly of polyhedral shells, including a deployable cube and a tetrahedron. Beyond geometric reconfiguration, the cube exhibits functional capabilities, including impact protection and multistable, partially deployed spatial configurations. Overall, this work establishes a fabrication‐friendly route to programmable and reprogrammable shape‐morphing that bridges local bistable interactions and system‐level structural functionality. The proposed design paradigm opens opportunities for reconfigurable mechanical metamaterials, protective enclosures, and scalable deployable structures inspired by grippers and architectural systems.

## 4 Experimental Section

### 4.1 Sample Fabrication

All experimental specimens were fabricated using Bambu TPU 90A filament (Bambu Lab) with a Raise3D E2 fused deposition modeling (FDM) printer. The printed TPU has an elastic modulus of E=8 MPa and a Poisson's ratio of ν=0.45. Prior to printing, the filament was dried at 70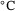 for 7 h. A 0.4 mm nozzle and a layer height of 0.1 mm were used. Printing was performed at a speed of 20 mm $s^{-1}$, with the build‐plate temperature set to 45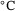 and the nozzle temperature to 223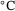.

### 4.2 Impact Protection and Deployable Architectural Demonstration

#### 4.2.1 Drop‐Impact Test

Impact protection was evaluated using a vertical drop test, as illustrated in Figure 5a. Pre‐folded origami cubes were placed on a rigid substrate, and a rigid impactor was released from a fixed height of $H_{0}=50$ cm. Three impactor masses, m=9.28, 21.06, and 30.28 g, were tested, corresponding to impact energies of 0.046, 0.103, and 0.149 J, respectively, estimated from E=mgh. The impact process was recorded using a camera, from which sequential frames were extracted for analysis (Figure 5b–d). For each mass, three repeated trials were conducted to quantify the drop‐impact response from the recorded videos. The folded origami cubes exhibited maximum compression displacements $\delta_{\max}$ of 1.27±0.05, 3.21±0.05, and 4.55±0.10 cm, respectively, demonstrating a measurable buffering stroke under increasing impact mass. The corresponding first‐bounce rebound heights $H_{r}$, height‐based apparent coefficients of restitution $e_{\mathrm{app}}$, and rebound‐energy‐loss proxies $\Delta E_{\mathrm{proxy}}$ are summarized in Table S1 and Figure S7. These metrics quantify the deformation and rebound response of the folded enclosure; the reported $\Delta E_{\mathrm{proxy}}$ is used as a system‐level rebound‐energy‐loss proxy rather than a direct measurement of the energy dissipated exclusively by the enclosure.

#### 4.2.2 Deployable Configurations

The reprogrammable folding capability was further demonstrated through partially deployed configurations of the origami cube (Figure 5e–g). Starting from the fully enclosed state, selected hinges were actuated to access intermediate stable configurations. The resulting structures remain self‐supporting and exhibit different accessible geometries depending on the underlying net design.

### 4.3 Modeling and Simulation

3D CAD models were created in SolidWorks 2024. FEM analyses were performed in Abaqus/Standard on the University of Birmingham BlueBEAR high‐performance computing (HPC) cluster. All cases were modeled using S4R shell elements and a consistent implicit dynamic quasi‐static procedure to capture snap‐through and post‐buckling responses. A three‐step loading and relaxation protocol was adopted. In Step 1, all boundary edges of the sheet were fully constrained, and a pressure load was applied to the dimple surfaces with a linear ramp to trigger inversion. In Step 2, the pressure load was removed while the edge constraints were maintained, allowing the structure to reach an equilibrium configuration. In Step 3, all boundary constraints were released to obtain the final free, relaxed deformed state. The material behavior was described using a compressible Neo‐Hookean hyperelastic model with parameters $C_{10}=1.4286$ MPa and $D_{1}=0.15$ MPa^‐1^. For all simulations, energy‐based convergence was checked to ensure stable solutions. Mesh convergence was verified by mesh‐independence studies before the parameter sweeps. The evolution of the total strain energy during the loading, constrained unloading, and final relaxation stages is provided for representative hinge‐level and system‐level simulations in Figure S8. The energy histories confirm that the reported post‐snap configurations correspond to relaxed states after complete removal of the applied pressure and boundary constraints.

## Conflicts of Interest

The authors declare no conflict of interest.

## Supporting information


**Supporting File 1**: advs76901‐sup‐0001‐SuppMat.pdf.

**Supporting File 2**: advs76901‐sup‐0002‐MovieS1.mp4.

**Supporting File 3**: advs76901‐sup‐0003‐MovieS2.mp4.

**Supporting File 4**: advs76901‐sup‐0004‐MovieS3.mp4.

**Supporting File 5**: advs76901‐sup‐0005‐MovieS4.mp4.

**Supporting File 6**: advs76901‐sup‐0006‐MovieS5.mp4.

**Supporting File 7**: advs76901‐sup‐0007‐MovieS6.mp4.

## Data Availability

The data that support the findings of this study are available from the corresponding author upon reasonable request.
