# Supplementary material for: Dimple‐Encoded Reprogrammable Origami
Source: Adv Sci (Weinh). 2026 Aug 5:e76901. Online ahead of print. doi: 10.1002/advs.76901 (PMC13440216; doi:10.1002/advs.76901)
Supplement: Supplementary file 1 [file ADVS-9999-e76901-s001.pdf]

# Supporting Information

## Dimple-Encoded Reprogrammable Origami

Qun Zhang[1], Weicheng Huang[2]*, Amir Hajiyavand[1], Hyunyoung Kim[3], Claire Dancer[4], Karl Dearn[1]*, Mingchao Liu[1]*

1. Department of Mechanical Engineering, University of Birmingham, Birmingham, B15 2TT, UK
2. School of Engineering, Newcastle University, Newcastle upon Tyne, NE1 7RU, UK
3. School of Computer Science, University of Birmingham, Birmingham B15 2TT, UK
4. School of Metallurgy and Materials, University of Birmingham, Birmingham B15 2TT, UK

Corresponding authors: *weicheng.huang@newcastle.ac.uk (W.H.); k.d.dearn@bham.ac.uk (K.D.); m.liu.2@bham.ac.uk (M.L.).

## S1 Bistability Screening and Interaction-driven Phase Diagrams

To ensure that programmable folding hinges are constructed from bistable dimple units, we first assess the stability of a single dimple using finite element method (FEM) simulations in Abaqus/Standard over a range of geometric parameters. Figure S1 shows the bistable and monostable regions in the $(R, h)$ design space. Based on this screening, all subsequent simulations in the main text and in the multi-column phase diagrams use a fixed geometry of $R = 5\,\mathrm{mm}$ and $h = 3.5\,\mathrm{mm}$.

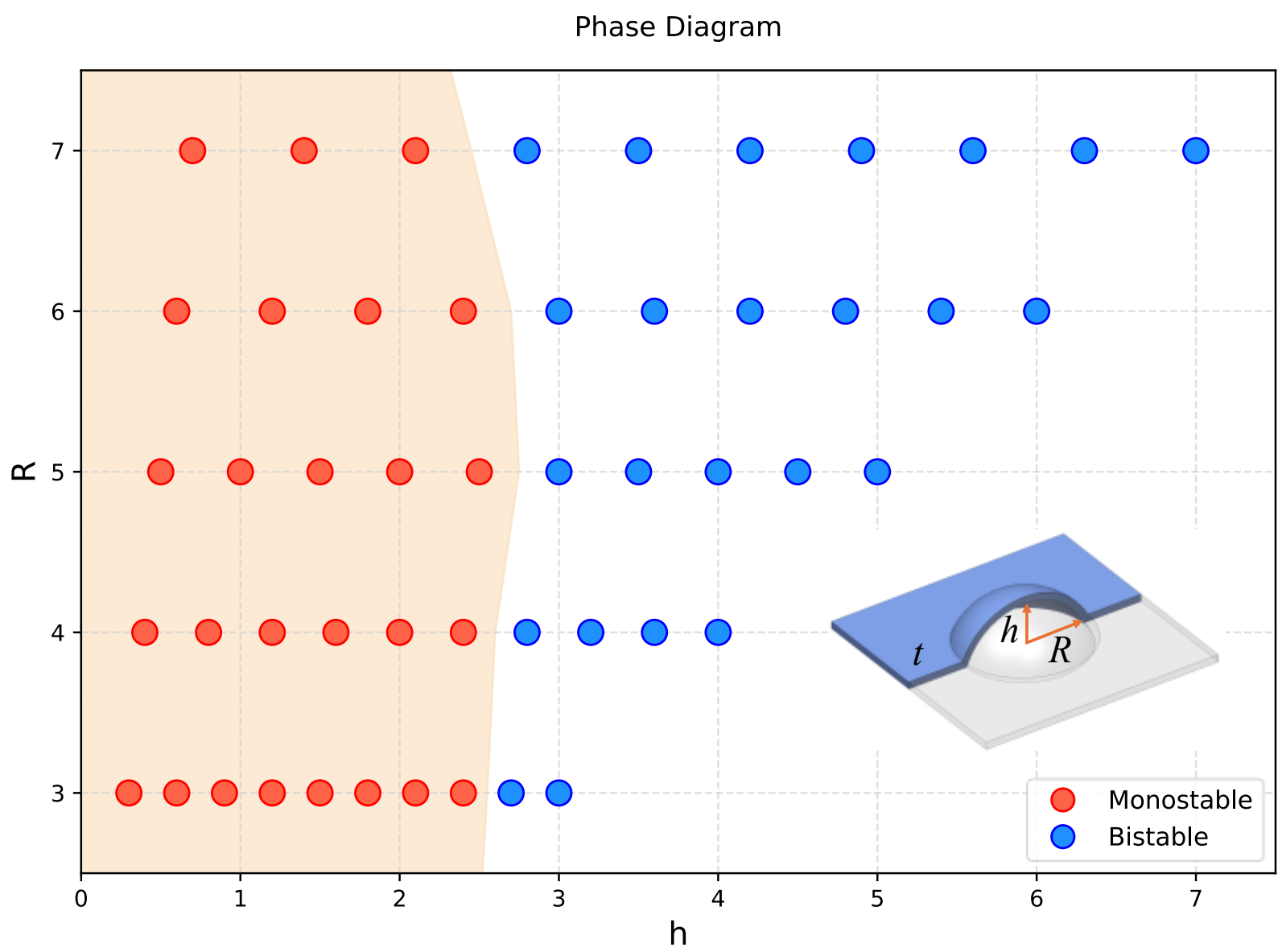


**Figure S1. Single-dimple bistability screening.** Bistability map obtained from FEM simulation as a function of dimple height $h$ and radius $R$ for a fixed sheet thickness $t$ = 0.6 mm. Red and blue markers denote monostable and bistable responses after dimple inversion and relaxation, respectively. The selected geometry, $R$ = 5 mm and $h$ = 3.5 mm, corresponding to $h/R = 0.7$, lies within the bistable domain and is used throughout the manuscript. The inset illustrates the geometric parameters $R$, $h$, and $t$.

Figure S2(a) provides additional insight into the interaction-guided phase behaviour discussed in the main text. We first consider a single-column dimple array aligned with a prescribed hinge line (red dashed line). Although snap-through of individual dimples generates localized deformation, a single column does not provide sufficient transverse coupling to concentrate curvature within

a narrow hinge band. Consequently, following inversion, the stored elastic energy is primarily released through boundary- and edge-dominated deformation, leading to global bending or warping of the sheet rather than a localized origami-like fold. This behaviour is observed across different longitudinal spacings $E_p$ (3.0, 4.0, and 6.5), where deformation spreads over the panel and relaxes through the free edges instead of forming a well-defined hinge.

Figure S2(b) shows how multi-column arrangements and topology variations enable hinge formation and deformation-mode selection. When dimples are distributed across multiple columns, overlapping boundary-layer fields enhance transverse interactions, promoting curvature localization and enabling selection of an effective hinge axis. Depending on the array topology and spacing, the system exhibits either a bending-dominated state with a hinge aligned with the prescribed direction (green marker), or a diagonal-bending state in which the effective hinge reorients along a diagonal (orange marker). This reorientation reflects interaction-driven mode competition, where the system selects the deformation pathway that minimizes the combined stretching and bending energy under the geometric incompatibility introduced by dimple inversion.

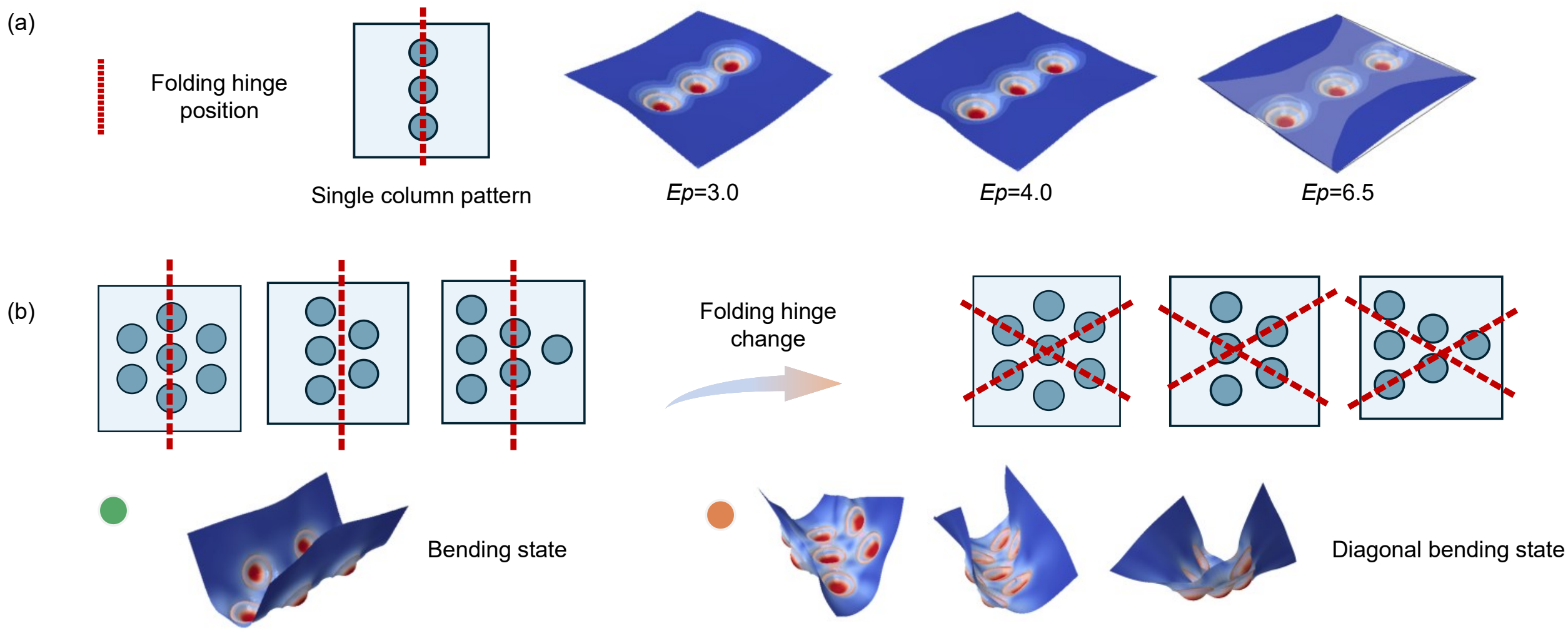


**Figure S2. Single-column limitation and deformation-mode selection in dimple-encoded folding.** (a) A single-column dimple array aligned with the prescribed hinge line (red dashed line) fails to produce a localized fold. After snap-through, deformation is dominated by global bending and edge effects rather than hinge-like rotation, as shown for different longitudinal spacings ($E_P$ = 3.0, 4.0, and 6.5). (b) Multi-column arrangements introduce transverse coupling, enabling reorientation of the effective hinge axis. Depending on the array topology, the structure exhibits either a bending-dominated state (green marker) or a diagonal bending state (orange marker), consistent with the morphing regimes in the phase diagram.

Overall, Figure S2 highlights two key design implications. First, a single-column arrangement is insufficient for robust hinge-like folding due to weak transverse coupling and dominant edge relaxation. Second, multi-column coupling enables hinge localization but introduces competing deformation modes, including diagonal bending, consistent with the phase-diagram regimes reported in the main text. These results motivate the use of multi-column arrays and interaction-guided selection of spacing and topology in the hinge design framework.

Figure S3 presents phase diagrams in the interaction-parameter space $(E_p, E_s)$ for three representative dimple-array topologies: 3–2, 3–2–1, and 2–3–2. Each point corresponds to a FEM simulation with the same baseline dimple geometry, while $E_p$ and $E_s$ control the normalized center and side spacings between neighbouring dimples. At small spacings (corresponding to low $E_p$ and $E_s$), the dimples interact strongly during snapping and cannot remain stably inverted after unloading. They therefore snap back, and the array tends to recover toward its original flat configuration,

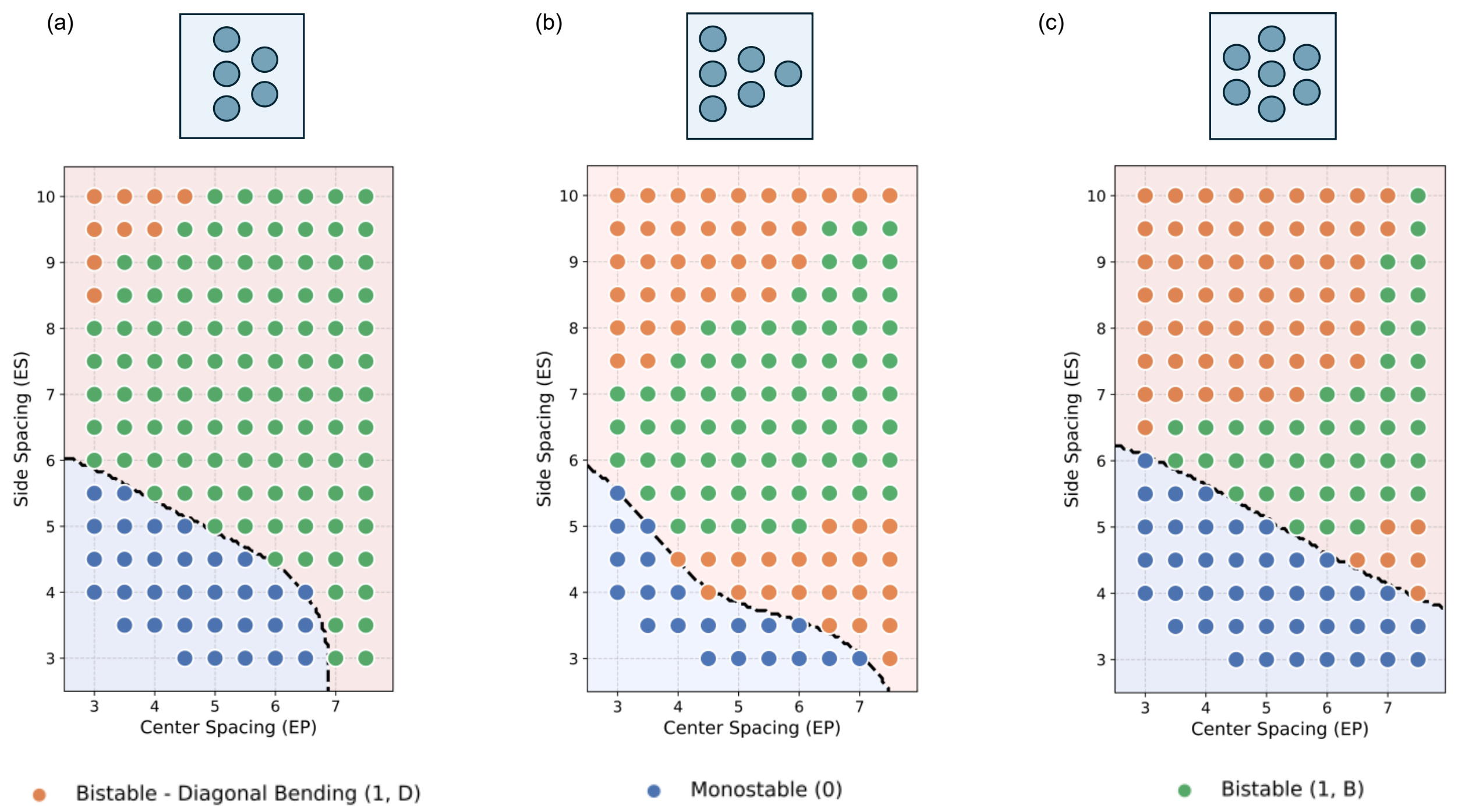


**Figure S3. Bistability screening and phase diagrams for representative multi-column dimple-array hinge topologies.** (a–c) Phase diagrams in the normalized spacing space $(E_p, E_s)$ for the 3–2, 3–2–1, and 2–3–2 dimple-array topologies, respectively. All simulations use the fixed baseline dimple geometry $R = 5$ mm, $h = 3.5$ mm, and $t = 0.6$ mm. Blue points denote monostable spring-back cases, where the dimples snap back after unloading. Orange and green points denote bistable cases, where the snapped dimples remain stable in the inverted state; orange points correspond to diagonal bending or mode switching, while green points correspond to well-defined hinge-like bending along the intended direction. The green regions define the validated design domain used for constructing the hinge-angle library.

leading to monostable spring-back behaviour at the system level. As the spacing increases, the interaction becomes less constrained, and the snapped dimples can remain stable in the inverted state, allowing the array to retain a stable post-snap morphing configuration and giving rise to bistable responses. Among these bistable cases, some exhibit diagonal bending or mode switching, whereas others form localized hinge-like bending along the intended direction. The dashed curves indicate the approximate boundaries between the monostable and bistable regimes for each topology. Only the well-defined hinge-bending cases are used to construct the hinge-angle library, while spring-back and diagonal-bending/mode-switching cases are excluded from the validated design domain.

## S2 Folding-Angle Measurement for Simulations and Experiments

To quantify hinge output robustly and enable direct comparison between simulations and experiments, we extract a two-dimensional deformed hinge profile and apply the same two-line Total Least Squares (TLS) fitting procedure in both cases. For simulations, the profile points are obtained from Abaqus node exports. For experiments, the profile points are extracted from specimen contours using the OpenCV-based workflow shown in Figure S4. In both cases, the folding angle is computed from the included angle between the fitted left and right edge directions.

Given a discrete profile

$$\{(x_i, y_i)\}_{i=1}^{N}, \tag{S1}$$

we optionally smooth the ordinate by a short moving-average filter to suppress high-frequency

numerical noise or contour jaggedness,

$$\tilde{y}_i = \frac{1}{w} \sum_{k=-m}^{m} y_{i+k}, \qquad w = 2m + 1, \tag{S2}$$

with endpoint padding for boundary indices. In the present datasets, $w = 1$ unless otherwise stated, so $\tilde{y}_i = y_i$ (no smoothing). This optional step is retained to improve robustness when profiles exhibit mesh- or image-induced oscillations. In the following, we denote $\mathbf{p}_i = (x_i, \tilde{y}_i)^\mathsf{T}$.

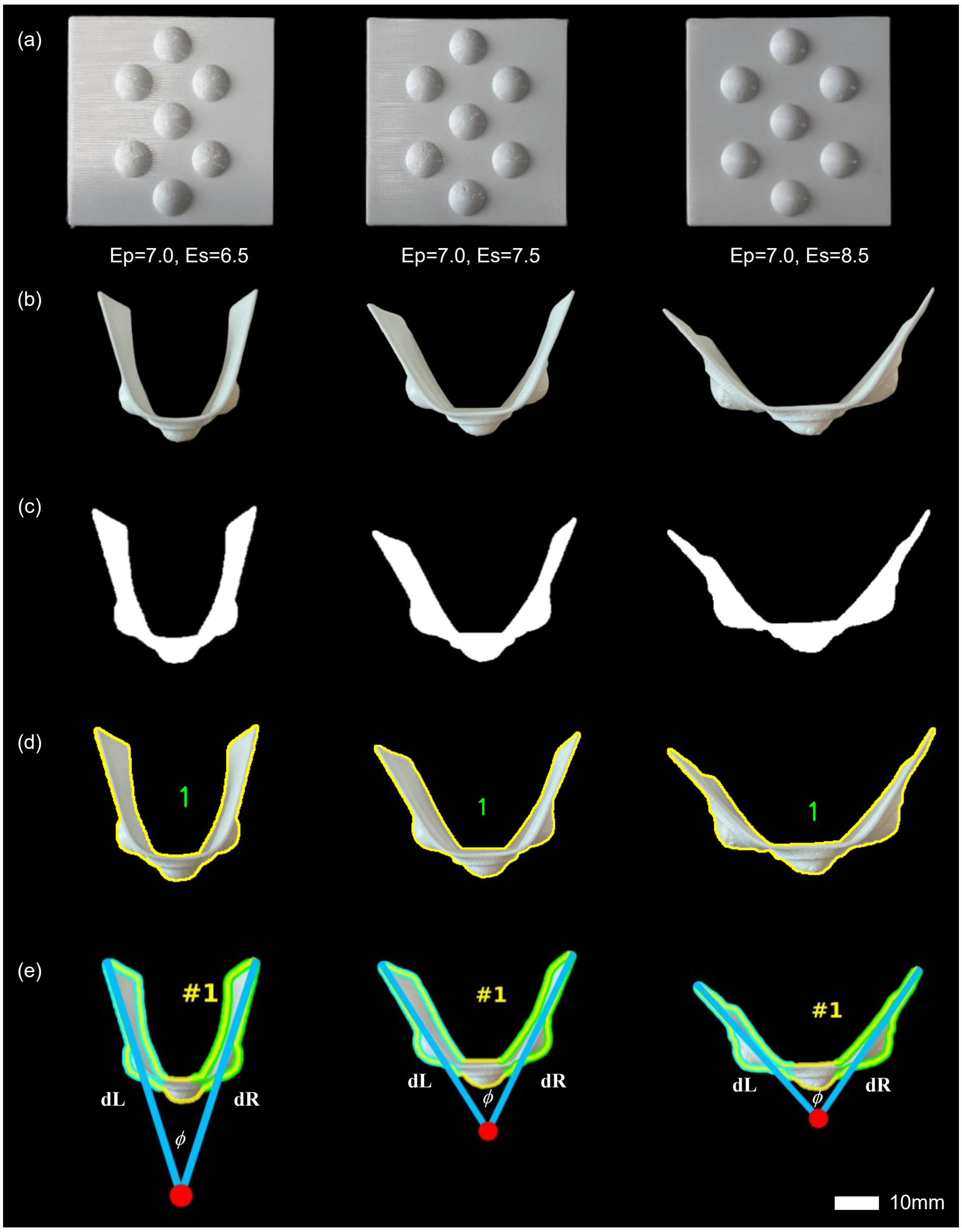


**Figure S4. OpenCV-based workflow for extracting folding angles from experiments.** (a) Experimental images of planar elastic sheets with different dimple configurations. (b) Deformed configurations after folding. (c) Grayscale conversion of the captured images. (d) Contour extraction of the deformed profiles using OpenCV. (e) Folding angles are computed from the extracted geometry by identifying the boundary and fitting the corresponding edge segments.

We model the hinge as two quasi-linear branches connected by a localized transition zone and identify the hinge using a two-line segmented fitting procedure. Candidate split indices $j$ are scanned along the profile while excluding a small buffer region around $j$ to avoid the highly curved transition zone, as well as buffer regions near both ends. For each candidate $j$, we define left and right point

sets $\mathcal{L}(j)$ and $\mathcal{R}(j)$ and fit a TLS line to each branch.

For a set of 2D points $\mathbf{p}_i = (x_i, \tilde{y}_i)^\mathsf{T}$ in one branch, the TLS line is represented by a centroid $\mathbf{c}$ and a unit direction $\mathbf{d}$:

$$\mathbf{c} = \frac{1}{n}\sum_{i=1}^{n} \mathbf{p}_i, \qquad \mathbf{d} = \arg\max_{\|\mathbf{d}\|=1} \sum_{i=1}^{n} \left(\mathbf{d}^\mathsf{T}(\mathbf{p}_i - \mathbf{c})\right)^2, \tag{S3}$$

which is given by the first principal direction of the centered data. The orthogonal distance from a point to the fitted line is

$$\delta_i = \left\| (\mathbf{I} - \mathbf{d}\mathbf{d}^\mathsf{T})(\mathbf{p}_i - \mathbf{c}) \right\|. \tag{S4}$$

The optimal split is selected by minimizing the mean squared TLS residual,

$$\mathcal{J}(j) = \frac{\sum_{i\in\mathcal{L}(j)} \delta_{i,\mathrm{L}}^2 + \sum_{i\in\mathcal{R}(j)} \delta_{i,\mathrm{R}}^2}{|\mathcal{L}(j)| + |\mathcal{R}(j)|}, \qquad j^* = \arg\min_j \mathcal{J}(j). \tag{S5}$$

The hinge vertex is defined as the intersection of the two fitted lines, obtained by solving

$$\mathbf{c}_\mathrm{L} + \alpha\, \mathbf{d}_\mathrm{L} = \mathbf{c}_\mathrm{R} + \beta\, \mathbf{d}_\mathrm{R} \tag{S6}$$

for $\alpha$ and $\beta$ (a $2 \times 2$ linear system). The included opening angle between the two fitted directions is then

$$\phi = \arccos(\eta), \qquad \eta = \frac{\mathbf{d}_\mathrm{L} \cdot \mathbf{d}_\mathrm{R}}{\|\mathbf{d}_\mathrm{L}\|\,\|\mathbf{d}_\mathrm{R}\|}, \tag{S7}$$

where $\eta$ is numerically clipped to $[-1, 1]$ before evaluating $\arccos(\cdot)$ to avoid floating-point issues.

In this work we report the folding angle as the supplementary angle

$$\theta = 180^\circ - \phi, \tag{S8}$$

so that larger $\theta$ corresponds to a deeper, more hinge-like fold (smaller opening), whereas smaller $\theta$ indicates a shallower fold (larger opening).

In Figure S4(e), the two fitted edge segments correspond to the TLS direction vectors $\mathbf{d}_\mathrm{L}$ and $\mathbf{d}_\mathrm{R}$. Their included angle defines $\phi$, from which $\theta = 180^\circ - \phi$ is obtained. Experimental and simulation folding angles are thus computed using the same geometric definition and fitting operator, differing only in the source of the profile points. Parameter sets that exhibit monostable spring-back are excluded from the angle design charts and summarized separately.

To quantify the experimental variability of the calibrated hinge-angle library, we fabricated replicate specimens for the three representative dimple-encoded hinge-band topologies used in the main text: 3–2, 3–2–1, and 2–3–2. The undeformed specimens and their corresponding folded configurations are shown in Figure S5(a–c), respectively. In each panel, the three rows correspond to ($E_s = 6.5$, 7.5, and 8.5), while the three columns show three independently fabricated and tested specimens for the same parameter set. For all cases, the longitudinal spacing was fixed at ($E_p = 6.5$), while the transverse spacing ($E_s$) was varied to examine its effect on the resulting hinge angle. Each parameter set was independently 3D printed, activated, and allowed to relax before imaging.

The deformed profiles were then processed using the same OpenCV-based contour extraction and TLS line-fitting workflow described above. The resulting folding angles were reported as mean values with standard-deviation error bars, thereby capturing the variability associated with fabrication, dimple activation, and post-snap-through relaxation. These experimental measurements are included in Figure 2d of the main text and compared with the FEM-based design charts. The

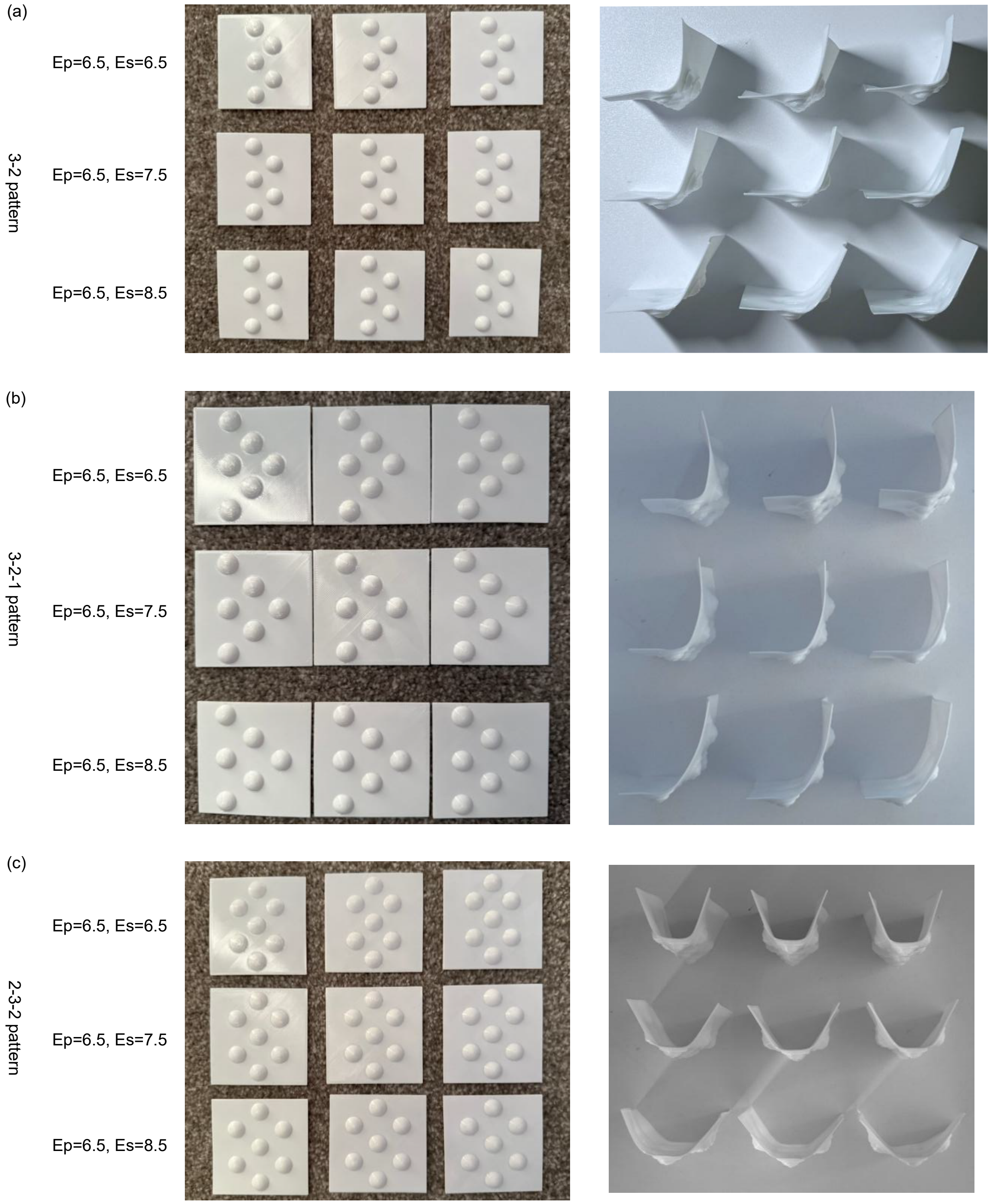


**Figure S5. Experimental validation of representative hinge-library designs.** Replicate 3D-printed specimens and corresponding deformed configurations for three representative hinge topologies: (a) 3–2 pattern, (b) 3–2–1 pattern, and (c) 2–3–2 pattern. For each topology, $E_p$ was fixed at 6.5, while $E_s$ was varied from 6.5 to 8.5. Each parameter set was fabricated and tested using three independent specimens. The left panels show the undeformed specimens before dimple activation, and the right panels show the folded configurations after snap-through and relaxation. The folding angles extracted using the OpenCV/TLS workflow are reported as mean values with standard-deviation error bars in Figure 2d.

agreement between the replicate experiments and the FEM trends supports the use of the calibrated design charts as a practical hinge-angle library within the validated bistable bending domain.

## S3 Activation-Path Independence of Representative Single-Hinge Patterns

To examine whether the final folded geometry depends on the order of dimple activation, we performed additional experiments on representative single-hinge patterns used in the hinge-angle library. For each pattern, the same set of target dimples was manually activated using different sequences, including simultaneous activation and sequential activation in different orders. After activation, the structure was allowed to relax freely.

As shown in Movies S1, different activation sequences lead to identical final folded configurations. This indicates that, within the robust bistable bending domain used to construct the hinge-angle library, the final folded state is primarily determined by the activated dimple set and the array geometry, rather than by the detailed triggering sequence.

## S4 Kirigami-Assisted Decoupling at Hinge Junctions

When multiple dimple-encoded hinges intersect within a compact junction region (e.g., in square-based nets), fully connected sheets exhibit parasitic out-of-plane warping and loss of planarity (Figure S6(a)). This behaviour arises from interaction between inverted dimples: each dimple generates a localized deformation field that decays over a boundary-layer length scale $\ell_b \sim \sqrt{tR}$. For densely packed hinge bands, these boundary layers overlap, and at junctions the superposition of adjacent fields imposes competing curvature and rotation on the same material region. This geometric incompatibility introduces in-plane stretching, which is energetically penalized relative to bending in thin sheets, leading to global bending, twisting, and local wrinkling or buckling instead of the intended hinge-like folding.

To mitigate this coupling, localized kirigami slits are introduced at junction regions (Figure S6(b)). These cuts act as mechanical release features that reduce the effective in-plane constraint area transmitting forces between neighboring hinge bands and permit relative rotation with reduced stretching. In a minimal energy framework, the junction coupling can be characterized by an in-plane stretching penalty $U_c \sim Y\,\varepsilon^2 A_c$, where $Y$ is the stretching modulus and $A_c$ is the effective coupled area. Introducing slits reduces $A_c$, relaxes compatibility constraints, and lowers $U_c$, thereby suppressing interaction-induced warping.

Kirigami is applied only near junctions where hinge boundary layers overlap, while the remainder of each face remains intact to preserve structural integrity. With kirigami, the sheet remains flatter prior to folding and exhibits more reliable and repeatable transformation into the target polyhedral geometry, indicating that hinge functionality is preserved while junction coupling is reduced.

## S5 Video-based Quantification of Impact-Buffering Response

To provide quantitative evidence for the impact-buffering demonstration of the dimple-encoded deployable origami cube, we performed drop-impact tests using three impactor masses, $m = 9.28$, 21.06, and 30.28 g, released from a fixed drop height of $H_0 = 50$ cm. For each impactor mass, three repeated trials were conducted ($n = 3$). From the recorded videos, we extracted two primary kinematic quantities: the maximum compression displacement of the origami cube, $\delta_{\max}$, and the first-bounce rebound height of the impactor, $H_r$.

The rebound height was measured relative to the same pre-impact reference plane used to define the release height $H_0$. Following the standard rebound-height method for vertical impact tests [1],

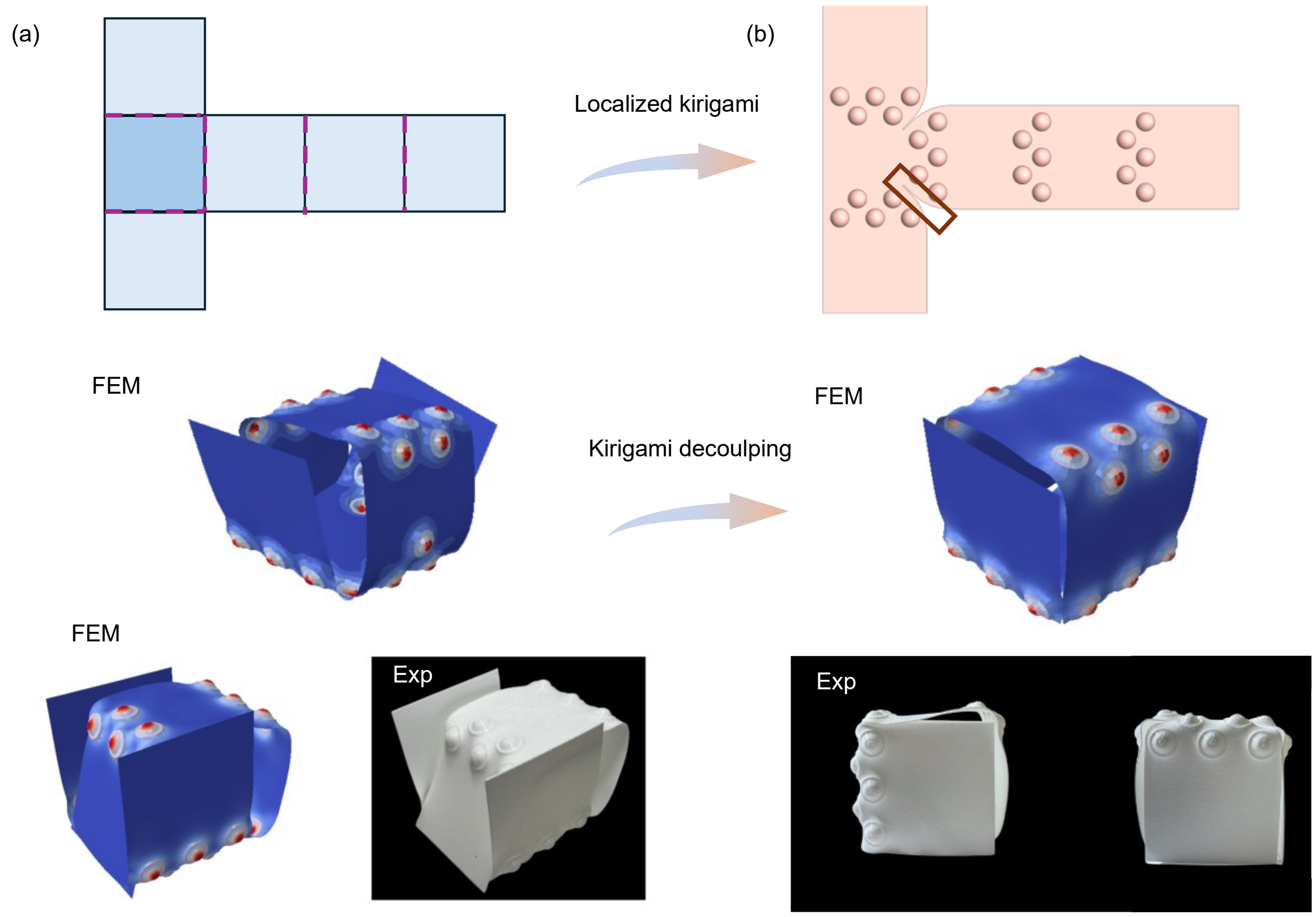


**Figure S6. Localized kirigami decoupling improves cube closure by mitigating dimple–dimple interactions at crowded junctions.** (a) Cube net without kirigami. Top: planar sheet with densely packed dimple arrays along the hinge network. Middle: finite element results after activation showing incomplete closure due to strong interactions at junctions. Bottom: corresponding FEM and experimental configurations exhibiting a residual gap. (b) Cube net with localized kirigami introduced near the junction (boxed). Middle: finite element results showing improved compatibility and complete closure after relaxation. Bottom: experimental realizations confirming reliable cube closure enabled by kirigami-induced decoupling.

we define the height-based apparent coefficient of restitution as

$$e_{\text{app}} = \sqrt{\frac{H_r}{H_0}}, \tag{S9}$$

where $H_r$ is the first-bounce rebound height. This expression follows from the ratio of rebound to incident velocities under a vertical ballistic-flight approximation. Because the origami cube undergoes finite deformation during impact, $e_{\text{app}}$ is interpreted as a system-level rebound metric for the impactor–origami-cube system, rather than as an intrinsic material coefficient of restitution.

We further define a system-level rebound-energy-loss proxy as

$$\Delta E_{\text{proxy}} = mg(H_0 - H_r), \tag{S10}$$

where $g$ is the gravitational acceleration. This quantity represents the portion of the impactor gravitational potential energy that is not recovered as rebound potential energy. It is therefore reported as a rebound-energy-loss proxy, rather than as a direct measurement of the energy dissipated exclusively by the origami cube.

The measured values are summarized in Table S1 and Figure S7. The rebound height $H_r$ and the apparent restitution $e_{\text{app}}$ show a non-monotonic dependence on impactor mass. For the

**Table S1. Quantitative drop-impact metrics extracted from video recordings (mean ± s.d., $n = 3$).**

| $m$ (g) | $H_0$ (cm) | $H_r$ (cm) | $\delta_{\max}$ (cm) | $e_{\text{app}}$ | $\Delta E_{\text{proxy}}$ (J) |
|---|---|---|---|---|---|
| 9.28 | 50 | $2.15 \pm 0.05$ | $1.27 \pm 0.05$ | $0.207 \pm 0.002$ | $0.04356 \pm 0.00005$ |
| 21.06 | 50 | $6.15 \pm 0.05$ | $3.21 \pm 0.05$ | $0.351 \pm 0.001$ | $0.09059 \pm 0.00010$ |
| 30.28 | 50 | $4.33 \pm 0.04$ | $4.55 \pm 0.10$ | $0.294 \pm 0.001$ | $0.13565 \pm 0.00012$ |

The values of $e_{\text{app}}$ and $\Delta E_{\text{proxy}}$ were calculated for each individual trial using Equations S9 and S10, respectively, before reporting the mean and standard deviation across three trials.

smallest impactor mass ($m = 9.28$ g), the origami cube undergoes only limited compression, with $\delta_{\max} = 1.27 \pm 0.05$ cm, indicating limited engagement of the programmed buffering deformation. For the intermediate mass ($m = 21.06$ g), the origami cube exhibits a larger but recoverable deformation, leading to the largest rebound height among the tested cases. For the largest impactor mass ($m = 30.28$ g), the origami cube reaches the greatest compression displacement, $\delta_{\max} = 4.55 \pm 0.10$ cm, indicating stronger engagement of the structural buffering mode. The lower rebound height compared with the intermediate-mass case is consistent with increased deformation of the origami cube and reduced recovery of rebound energy.

Accordingly, $H_r$ and $e_{\text{app}}$ are reported as video-extracted dynamic response metrics and should not be interpreted as a simple monotonic ranking of impact-buffering performance. Similarly, because $\Delta E_{\text{proxy}}$ explicitly depends on the impactor mass, comparisons between different masses should be interpreted together with $\delta_{\max}$ and the rebound response. Direct measurement of impact force would require an instrumented impactor or force sensor; therefore, the present proof-of-concept setup focuses on video-based metrics that can be consistently extracted from the recorded experiments.

## S6 Energy Evolution During Snap-through and Relaxation in FEM Simulations

To provide quantitative evidence for the snap-through and post-snap relaxation captured by our FEM simulations, we report the global strain energy as a function of analysis step time for two representative cases (Figure S8). The simulations follow the three-step protocol used throughout this work: (Step 1) boundary edges are constrained and a linearly ramped pressure is applied to the target dimple surfaces to trigger inversion; (Step 2) the pressure is removed while the boundary constraints are maintained, allowing the structure to settle into a constrained post-snap equilibrium; and (Step 3) all boundary constraints are released and the structure is further relaxed to reach the final free equilibrium configuration.

Figure S8 shows that the strain energy increases during the loading stage as elastic energy is stored while the dimples are driven toward snap-through. Upon pressure removal with fixed boundaries, the strain energy decreases substantially as the system relaxes into a post-snap configuration. After full boundary release, the strain energy converges to a nearly constant plateau, indicating that the simulated structure has reached a stable relaxed equilibrium state. The same qualitative energy evolution is observed for both a local hinge patch with a 2–3–2 pattern (Figure S8(a)) and a multi-hinge strip programmed to be folded into an equilateral triangle (Figure S8(b)). This supports that the final shapes reported in the manuscript correspond to relaxed equilibria rather than transient, load-supported configurations.

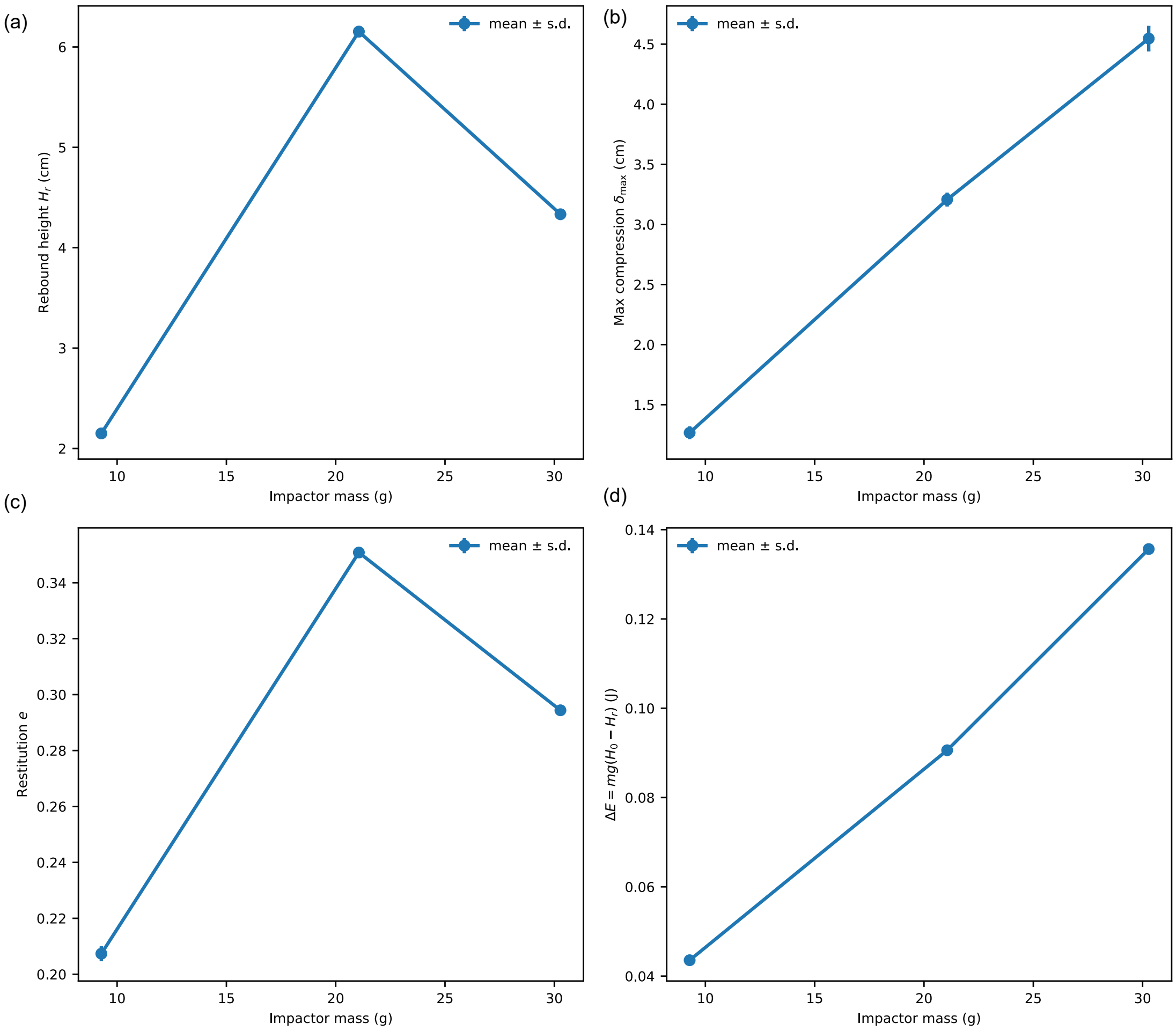


**Figure S7. Video-based quantitative impact metrics.** Impact metrics extracted from video recordings for repeated drop tests at a fixed drop height of $H_0 = 50$ cm. (a) First-bounce rebound height $H_r$ as a function of impactor mass. (b) Maximum compression displacement $\delta_{\text{max}}$ as a function of impactor mass. (c) Height-based apparent coefficient of restitution, defined as $e_{\text{app}} = \sqrt{H_r/H_0}$. (d) System-level rebound-energy-loss proxy, defined as $\Delta E_{\text{proxy}} = mg(H_0 - H_r)$. Data are reported as mean ± s.d. over three repeated trials ($n = 3$). The rebound height and apparent restitution vary non-monotonically with impactor mass, whereas the maximum compression increases monotonically as the impactor becomes heavier. The rebound-energy-loss proxy increases over the tested mass range, reflecting both its explicit dependence on impactor mass and the increased structural deformation under heavier impacts.

# S7 Supplementary Videos

Movie S1 (.mp4 format). Experimental validation of activation-path independence for a representative dimple-encoded hinge pattern.

Movie S2 (.mp4 format). FEM simulation of a multi-hinge dimpled strip folding into a triangular configuration.

Movie S3 (.mp4 format). Experimental reconfiguration of a single 3D-printed dimpled strip into triangular, square, and pentagonal configurations.

Movie S4 (.mp4 format). FEM simulations of flat-to-3D folding from planar dimpled nets into a tetrahedron and a cube.

Movie S5 (.mp4 format). Experimental flat-to-3D folding of 3D-printed dimpled planar nets into a tetrahedron and a cube.

Movie S6 (.mp4 format). Experimental drop-impact response of the pre-folded origami cube under impacts from three different masses.

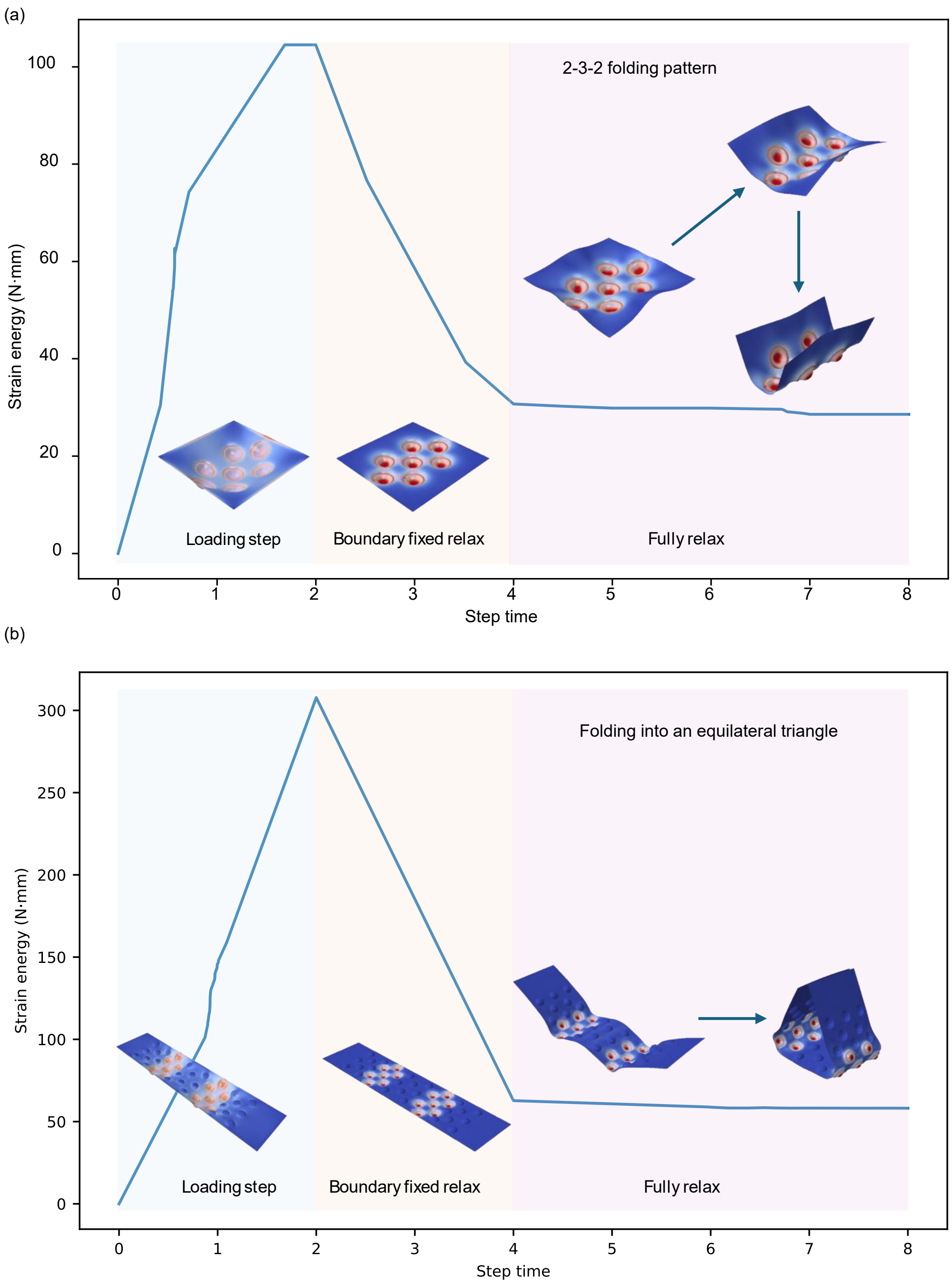


**Figure S8. Strain-energy evolution during representative snap-through simulations.** Total strain energy as a function of step time for (a) a dimpled sheet with a 2–3–2 hinge pattern and (b) a multi-hinge strip programmed to fold into an equilateral triangle. The shaded regions indicate the three-step Abaqus/Standard protocol: loading with pressure applied to the dimples, boundary-fixed relaxation after pressure removal, and full relaxation after releasing the boundary constraints. Representative snapshots show the corresponding deformation states. In both cases, the strain energy increases during loading, decreases during relaxation, and eventually reaches a plateau, indicating convergence to a stable post-snap configuration.

## References

[1] L. J. Briggs, Methods for Measuring the Coefficient of Restitution and the Spin of a Ball, *Journal of Research of the National Bureau of Standards* **34** (1945), 1–23.
